# Intestinal Barrier Damage and Growth Retardation Caused by Exposure to Polystyrene Nanoplastics Through Lactation Milk in Developing Mice

**DOI:** 10.3390/nano15010069

**Published:** 2025-01-04

**Authors:** Chaoyu Zhou, Haiyan Wu, Lei Zhang, Xiao Xiao, Xiaodan Wang, Mingju Li, Runqiu Cai, Jia You, Qi Chen, Yifei Yang, Xinyuan Tian, Qianyu Bai, Yinzhu Chen, Huihui Bao, Tianlong Liu

**Affiliations:** 1 National Key Laboratory of Veterinary Public Health and Safety, College of Veterinary Medicine, China Agricultural University, Beijing 100193, China; cvmzhouchaoyu@cau.edu.cn (C.Z.); wuhaiyan_linda@sina.com (H.W.); 18615110157@163.com (R.C.); baiyi@cau.edu.cn (Y.Y.); tianxy@cau.edu.cn (X.T.); 15046900669@163.com (Q.B.); chenyinzhu827@163.com (Y.C.); 2 Chinese Academy of Medical Science Research Unit, NHC Key Laboratory of Food Safety Risk Assessment, China National Center for Food Safety Risk Assessment, Beijing 100022, China; zhanglei@cfsa.net.cn (L.Z.); xiaoxiao@cfsa.net.cn (X.X.); wangxiaodan@cfsa.net.cn (X.W.); 3 Yantai Animal Disease Control Center, Yantai 264003, China; limingju521@sina.cn; 4 Yantai Agricultural Technology Extension Center, Yantai 264001, China; youconghua@163.com; 5 Livestock and Veterinary Development Center of Zoucheng, Hong Kong, China; zcxcpk@126.com

**Keywords:** microplastics, polystyrene nanoplastics, developmental mice, intestinal toxicity

## Abstract

Microplastics, defined as plastic fragments smaller than 5 mm, degrade from larger pollutants, with nanoscale microplastic particles presenting significant biological interactions. This study investigates the toxic effects of polystyrene nanoplastics (PS-NPs) on juvenile mice, which were exposed through lactation milk and drinking water at concentrations of 0.01 mg/mL, 0.1 mg/mL, and 1 mg/mL. The results show that PS-NP exposure during lactation and juvenile periods caused delayed weight gain and impaired organ development, particularly in the liver and kidneys, without causing functional abnormalities or toxic injuries. The primary toxicity of PS-NPs was observed in the intestinal tract, including shortened villi, disrupted tight junctions, inhibited epithelial cell proliferation, and oxidative stress responses. These findings highlight the importance of evaluating the developmental toxicity of nanoplastics at environmentally relevant doses.

## 1. Introduction

The persistent presence of microplastics (MPs) and nanoplastics (NPs) in the environment poses significant risks to public health due to their stable physicochemical properties and ability to accumulate over time [1]. Due to its high tensile strength, swelling ability, stable chemical properties, regeneration characteristics, large surface area, and economic feasibility, polystyrene (PS) is widely used in various products [2]. However, these plastic particles pose a threat to the environment and human health. On one hand, direct human contact with PS is a potentially critical issue. This contact can occur through various sources, such as food packaging materials, containers, personal care products, biomedical products, and disposable water bottles [3,4]. Additionally, PS particles may also accumulate in the environment through the food chain, leading to damage to animal health. Studies have shown that the ingestion of microplastics in the food chain results in adverse effects, particularly in marine invertebrates [5,6]. For instance, oysters significantly increased their consumption of microalgae and nutrient absorption when exposed to polystyrene microbeads [7].

For humans, nanoplastics are primarily ingested via the oral route through drinking water and food [8,9,10]. It is estimated that humans consume roughly 398–431 g of microplastics from food annually, with additional intake from drinking water [11]. However, there is significant variability in these estimates, and one review suggested that the minimum estimated weight of microplastics ingested per person is 33.2 g [12]. The wide range of reported values is largely attributed to current limitations in microplastic detection methodologies, including the lack of standardized protocols for sample preparation and analytical tools. As a result, the exact level of human exposure to microplastics from the environment, diet, and drinking water remains inconclusive. In this study, the maximum and minimum exposure levels reported in the existing literature are converted using the body surface area normalization method and subsequently used to establish high-dose (1 mg/mL) and medium-dose (0.1 mg/mL) groups for experiments. One major concern in studies on MPs and NPs is the use of exposure doses which far exceed realistic environmental levels, making the results difficult to translate to actual biological exposure scenarios [13]. Therefore, a lower-order magnitude dose group is established as the low-dose group (0.01 mg/mL) in this study. Furthermore, gavage administration necessitates the restraint of conscious or lightly anesthetized animals, a procedure which is known to trigger a stress response, including elevated plasma corticosterone levels, in both rats and mice [14].

Concerns have also been raised about the reproductive toxicity of polystyrene nanoparticles (PS-NPs), particularly their effects on offspring development [15,16]. Studies have shown that gestational exposure to polystyrene particles can cause anxiety-like behaviors in progeny, reduced gamma-aminobutyric acid (GABA) levels, delayed retinal development, and disturbed neurotransmitter metabolism [17]. PS-NPs have also been linked to neural tube defects via impaired apoptotic cell death and disrupted autophagy, suggesting transgenerational toxic effects which impact growth and development [18].

Therefore, this study aims to investigate whether maternal exposure to PS-NPs through drinking water induces toxic effects in offspring consuming breast milk during the lactation period and whether exposure to PS-NPs through drinking water during the developmental stage affects early growth and development in offspring. To address these issues, this study uses lower doses of PS-NPs administered via drinking water with assessments conducted on two observation time points, namely post-delivery days 18 and 46, to evaluate the impact of PS-NP exposure on early growth and development in offspring mice.

## 2. Materials and Methods

### 2.1. Production and Characterization of PS-NPs

PS-NPs were synthesized in our laboratory using emulsion polymerization under anaerobic conditions to produce 100 nm particles. A total of 267 mL of distilled water was added to a three-neck flask and heated in an 80 °C water bath. Nitrogen gas was introduced for 30 min, after which 10 mL of styrene (Macklin^®^, Shanghai, China) and 1 mL of methylacrylic acid (Aladdin^®^, Shanghai, China) were slowly added while stirring at 800–1000 rpm for 30 min. Next, 0.15 g of potassium persulfate dissolved in 3 mL of water was added, and stirring continued under nitrogen flow for 50 min. The reaction was maintained in the water bath for 10–12 h, followed by 12 h of cooling to allow the evaporation of unreacted monomers. The resulting PS-NP suspension was dialyzed for 24 h. For concentration determination, 1 mL of PS-NP solution was dried and weighed to calculate the nanoparticle concentration. Rhodamine B (1 mg/100 mL) was added to the reaction system to produce PS-NPs with red fluorescence.

For morphological analysis, the PS-NP solution was diluted and dropped onto an electron microscopy grid coated with tin foil. After natural drying, the sample was sputter-coated and imaged using a scanning electron microscope (SEM, ZEISS GeminiSEM 300, ZEISS, Oberkochen, Germany). To determine the hydration particle size and zeta potential, the PS-NPs were diluted with distilled water, sonicated for uniform dispersion, and analyzed using dynamic light scattering (DLS; Malvern Zetasizer Nano ZS90, Malvern, UK) to measure the size distribution and zeta potential. For Fourier transform infrared spectroscopy (FTIR) analysis, 5–10 mL of PS-NP solution was frozen at −80 °C and freeze-dried for 8 h. The resulting powder was pressed into pellets and analyzed with FTIR (Thermo Fisher Scientific Nicolet iS20, Waltham, MA, USA) over a wavelength range from 500 to 4000 ${\mathrm{cm}}^{-1}$ to determine the chemical structure.

### 2.2. Animals and Treatment

Sixteen 8 week-old pregnant ICR female mice were purchased from SPF Biotechnology Co., Ltd. (Beijing, China) and randomly divided into four groups (n = 4 per group). Starting from post-delivery day 0 (PDD 0), the mice were provided with drinking water containing 0.01, 0.1, or 1 mg/mL of PS-NPs (low-, medium-, and high-dose groups), while the control group received distilled water. Weaning occurred on PDD 18, after which the maternal mice were euthanized, and 10 offspring per group were randomly selected for weight measurement and tissue collection. Tissue samples (heart, liver, spleen, lung, kidney, and small intestine) were weighed and fixed for lactation data analysis.

The remaining offspring (5 males and 5 females per group) were raised until PDD 46, when their weights were recorded, and they were euthanized. (Thus, some litters contributed more than two animals per dam.) Serum was collected for biochemical analysis, and tissue samples were processed similarly to the earlier group. Intestinal tissues were stored in liquid nitrogen and transferred to a −80 °C freezer for further analysis. All animal procedures were approved by the Institutional Animal Care and Use Committee of China Agricultural University (AW81503202-2-1).

### 2.3. Histological Analysis

Following complete fixation of the mouse tissue samples, the tissue blocks were subjected to a series of processing steps including dehydration, embedding, sectioning, and hematoxylin-eosin (H&E) staining. The detailed procedures for tissue dehydration and H&E staining are outlined in the Supplementary Materials. After staining, the slides were covered with neutral resin and allowed to air dry before examination under a microscope.

### 2.4. X-Ray Analysis of the Tibias and Femurs in the Offspring Mice

At the time of euthanasia, the right hind limb of each mouse was detached at the hip joint. X-ray imaging of the mice was performed using a high-resolution in vivo X-ray imaging system (M-20, USA). The imaging system was set to a voltage of 40 kV for an optimal resolution. The exposure time was set to 10 s per image. The length of the tibia and femur was then measured using Image J for analysis.

### 2.5. Preparation of Frozen Sections

The tissues used in this study were placed onto a tissue holder and embedded in optimal cutting temperature compound (OCT). Subsequently, the samples were rapidly frozen to −20 °C and secured onto a chuck, and 10 μm sections were cut using a Leica CM1950 freezing microtome (Heidelberg, Germany). The cryosections were then mounted onto glass slides and sealed with 4′,6-diamidino-2′-phenylindole (DAPI). Finally, the sections were imaged using a Leica fluorescence microscope (Heidelberg, Germany) within 30 min.

### 2.6. Immunohistochemistry

The blank paraffin sections were processed as follows. They were deparaffinized and rinsed three times with distilled water for 5 min each. Antigen retrieval was performed in 0.01 M sodium citrate buffer at 100 °C for 10 min, followed by natural cooling. The sections were then washed three times with PBS buffer for 5 min each. A 3% hydrogen peroxide solution was added, and the sections were incubated in the dark for 30 min, followed by three additional PBS washes of 5 min each. Goat serum was applied for blocking for 30 min, and the sections were incubated overnight at 4 °C with the primary antibody diluted according to the manufacturer’s instructions. Subsequently, they were washed three times with PBS buffer for 5 min each. Enzyme-conjugated secondary antibody was added, and the sections were incubated at room temperature for 30 min before being washed three times with PBS buffer for 5 min each. Development was performed using diaminobenzidine (DAB) chromogen. The sections were counterstained with hematoxylin for 2 min, dehydrated, cleared, and mounted. ZO-1 antibody (Proteintech^®^ 21773-1-AP, Rosemont, IL, USA) was used at a dilution of 1:3000, the β-actin antibody (Proteintech^®^ 66009-1-Ig) was used at a dilution of 1:20,000, and the PCNA antibody (Proteintech^®^ 60097-1-Ig) was used at a dilution of 1:5000.

### 2.7. Western Blotting

From each group, 10 mg of intestinal tissue was taken and added to efficient radio-immunoprecipitation assay (RIPA) tissue cell lysis buffer containing the protein protector phenylmethanesulfonyl fluoride (PMSF) for grinding. The mixture was centrifuged at 12,000 rpm for 10 min, and the supernatant was collected. Protein concentrations were determined using a bicinchoninic acid assay (BCA) kit (Solarbio PC0020, Beijing, China) in an ice environment and adjusted to uniform concentrations across all groups. The protein solution was mixed with sample buffer for gel electrophoresis (Bio-rad, Hercules, CA, USA), followed by heat treatment according to standard laboratory protocols. After 1 h of blocking with 5% non-fat milk at 4 °C, the primary antibodies were applied and left to incubate overnight. Subsequently, the secondary antibodies were applied for 1 h, followed by imaging in a Tanon 5200 exposure unit (Shanghai, China). The ZO-1 antibody (Proteintech^®^ 21773-1-AP) was diluted at a 1:3000 ratio. The β-actin antibody (Proteintech^®^ 66009-1-Ig) was diluted at a 1:20,000 ratio. The PCNA antibody (Proteintech^®^ 60097-1-Ig) was diluted at a 1:5000 ratio for WB.

### 2.8. Measurement of Oxidative Stress Markers in Intestinal Tissue

For superoxide dismutase (SOD) measurement, 10 mg of small intestinal tissue was taken and added to the SOD sample preparation buffer provided by the assay kit (Beyotime S0101S, Shanghai, China). The tissue was homogenized in a tissue grinder. After low-temperature centrifugation at 12,000× *g* for 5 min at 4 °C, the supernatant was collected. The protein concentration was determined using a BCA protein assay kit (Solarbio PC0020, Beijing, China), and the concentrations across the groups were adjusted to be consistent. The subsequent steps were carried out according to the manufacturer’s instructions. The absorbance was measured at 450 nm using a microplate reader. The inhibition percentage was calculated using the following formula:

$$\mathrm{Inhibition}\;\mathrm{Percentage}=\frac{\left[\left(A_{\mathrm{blank}\;\mathrm{control}\;1}-A_{\mathrm{blank}\;\mathrm{control}\;2}\right)-\left(A_{\mathrm{sample}}-A_{\mathrm{blank}\;\mathrm{control}\;3}\right)\right]}{\left(A_{\mathrm{blank}\;\mathrm{control}\;1}-A_{\mathrm{blank}\;\mathrm{control}\;2}\right)}\times 100\%$$

For the total glutathione (GSH) measurement, 20 mg of small intestinal tissue was taken and added to the sample homogenization buffer provided by the assay kit (Beyotime S0052, Shanghai, China). The tissue was homogenized using a tissue grinder. After low-temperature centrifugation at 10,000× *g* for 10 min at 4 °C, the supernatant was collected. Subsequently, the following steps were performed according to the manufacturer’s instructions. The absorbance within the range of 405–414 nm was measured using a microplate reader. A standard curve was constructed based on the absorbance values obtained from standards of different concentrations. The total glutathione content in the samples was then calculated by comparing their absorbance with the standard curve.

### 2.9. Serum Biochemical Parameter Analysis

The levels of serum albumin (ALB), cholesterol (CHOL), alkaline phosphatase (ALP), alanine aminotransferase (ALT), creatinine (CREA), and urea (UREA) in the PDD46 offspring mice were measured using an automatic biochemical analyzer (VetTest 8008, Idexx, Westbrook, ME, USA). Blood samples were collected via the orbital sinus, allowed to clot at room temperature for 30 min, and centrifuged at 3000× *g* for 15 min at 4 °C to separate the serum. The obtained serum was stored at −80 °C until analysis.

### 2.10. Statistical Analysis

All experimental data are expressed as the mean ± standard deviation. Data analysis was performed using GraphPad Prism 8. One-way analysis of variance (one-way ANOVA) was used to test for differences between the groups. A *p* value of <0.05 was considered statistically significant, and a *p* value of <0.01 was considered highly statistically significant.

## 3. Results

### 3.1. Characterization of PS-NPs

We characterized the synthesized PS-NPs. The average hydrodynamic diameter of the PS-NPs was 165 ± 38.07 nm, with a zeta potential of −34.9 ± 9.49 mV, indicating good dispersion in water. The Fourier transform infrared (FTIR) spectrum showed a C-H stretching vibration peak in the 3000–3100 ${\mathrm{cm}}^{-1}$ range, corresponding to the benzene ring CH=CH2 stretching vibration. Two peaks at 2923 ${\mathrm{cm}}^{-1}$ and 2851 ${\mathrm{cm}}^{-1}$ in the 2800–3000 ${\mathrm{cm}}^{-1}$ range corresponded to the asymmetric and symmetric vibrations of -CH2-. Peaks at 1601, 1493, and 1452 ${\mathrm{cm}}^{-1}$ were due to the benzene ring CH=CH- bending vibrations. Peaks at 756 and 698 ${\mathrm{cm}}^{-1}$ correspond to the out-of-plane bending of a monosubstituted benzene ring =CH. The most characteristic peaks of polystyrene are in the 3000–3100 ${\mathrm{cm}}^{-1}$ and 1450–1600 ${\mathrm{cm}}^{-1}$ ranges, with secondary characteristic peaks at 756 ${\mathrm{cm}}^{-1}$ and 698 ${\mathrm{cm}}^{-1}$. When comparing the FTIR spectrum of our synthesized PS-NPs with the standard spectrum library data, the results suggest high similarity to the standard polystyrene material, with a similarity of 90.95%.

### 3.2. Impact of PS-NPs on Body Weight Gain in Offspring Mice

**Figure 1:**
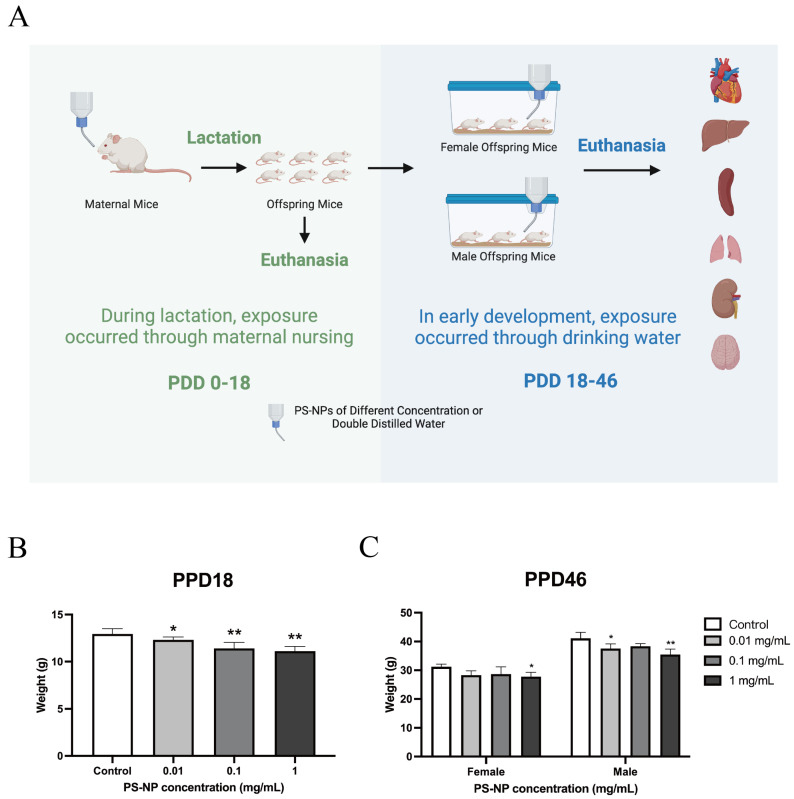
(**A**) Schematic diagram of toxicity experiment in mice. (**B**,**C**) Weight changes of offspring mice of PDD18 and PDD46 after exposure to different doses of PS-NPs. Note: Compared with the control group, * *p* < 0.05; ** *p* < 0.01. Data were analyzed using one-way ANOVA and are expressed as mean ± SD (n = 10 for (**B**), and n = 5 for (**C**)).

The mice were exposed to various concentrations of PS-NPs through lactation and drinking water from birth (Figure 1A). The characterization of the PS-NPs used in this study is detailed in Supplementary Figure S1. Body weights were measured on post-delivery days 18 (PDD18) and 46 (PDD46), as shown in Figure 1B,C. At PDD18, the average body weight of the low-dose group was reduced by 0.62 g (4.8%) compared with the control group, with a significant difference (*p* < 0.05). The medium- and high-dose groups exhibited more pronounced reductions, with average decreases of 1.54 g (11.9%) and 1.83 g (14.1%), respectively, showing highly significant differences compared with the control group (*p* < 0.01). At PDD46, the female offspring exposed to low and high doses of PS-NPs had their body weights reduced by 2.91 g (9.3%) and 3.45 g (11.1%), respectively, with significant differences (*p* < 0.05). In the medium-dose group, the body weight was reduced by 2.53 g (8.1%) compared with the control, but this difference was not significant. Male offspring exposed to low doses had their body weights reduced by 3.51 g (8.5%), showing a significant difference (*p* < 0.05), while high-dose exposure resulted in a reduction of 5.61 g (13.7%), with a highly significant difference (*p* < 0.01). The medium-dose group showed a weight reduction of 2.75 g (6.7%), but this was not significant. Overall, these findings indicate that PS-NP exposure through drinking water leads to reduced weight gain in developing mice, with no significant gender differences in the impact on weight gain.

### 3.3. Impact of PS-NPs on Organ and Skeletal Development in PDD18 Offspring Mice

**Figure 2:**
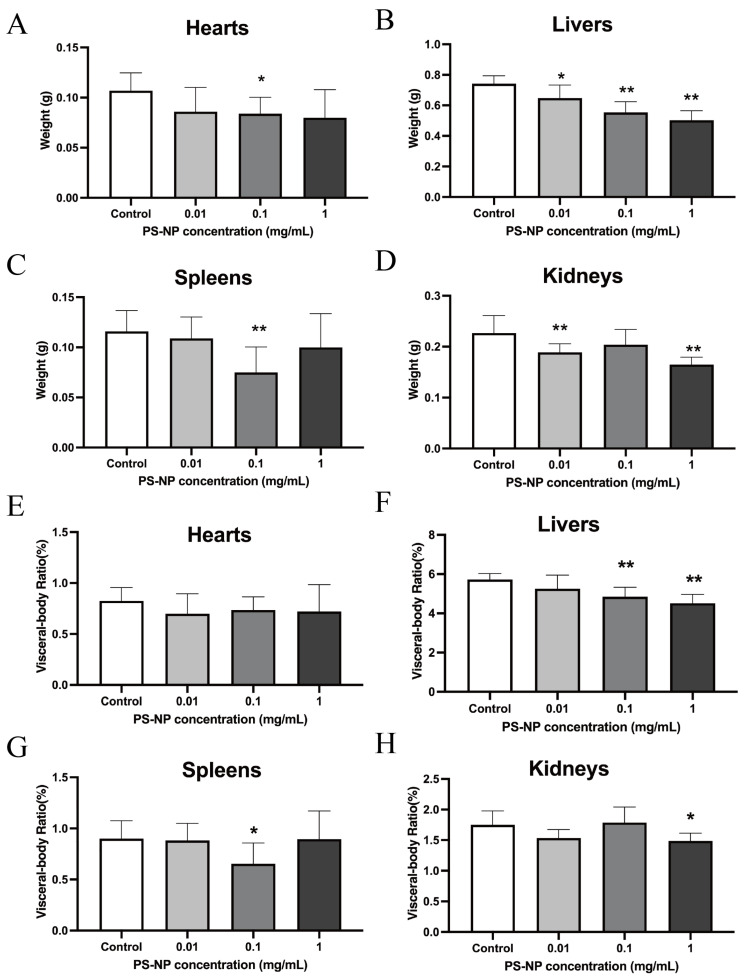
The effects of PS-NP exposure on the organs of the PDD18 (lactation period) offspring mice. (**A**–**D**) Changes in the main organ weights of the offspring mice at PDD18 after exposure to different doses of PS-NPs. Note: Compared with the control group, * *p* < 0.05; ** *p* < 0.01. (**E**–**H**) Changes in the main organ visceral body ratio in the offspring mice of PDD18 after exposure to different doses of PS-NPs. Note: Compared with the control group, * *p* < 0.05; ** *p* < 0.01. Data were analyzed using one-way ANOVA and expressed as mean ± SD (n = 10).

**Figure 3:**
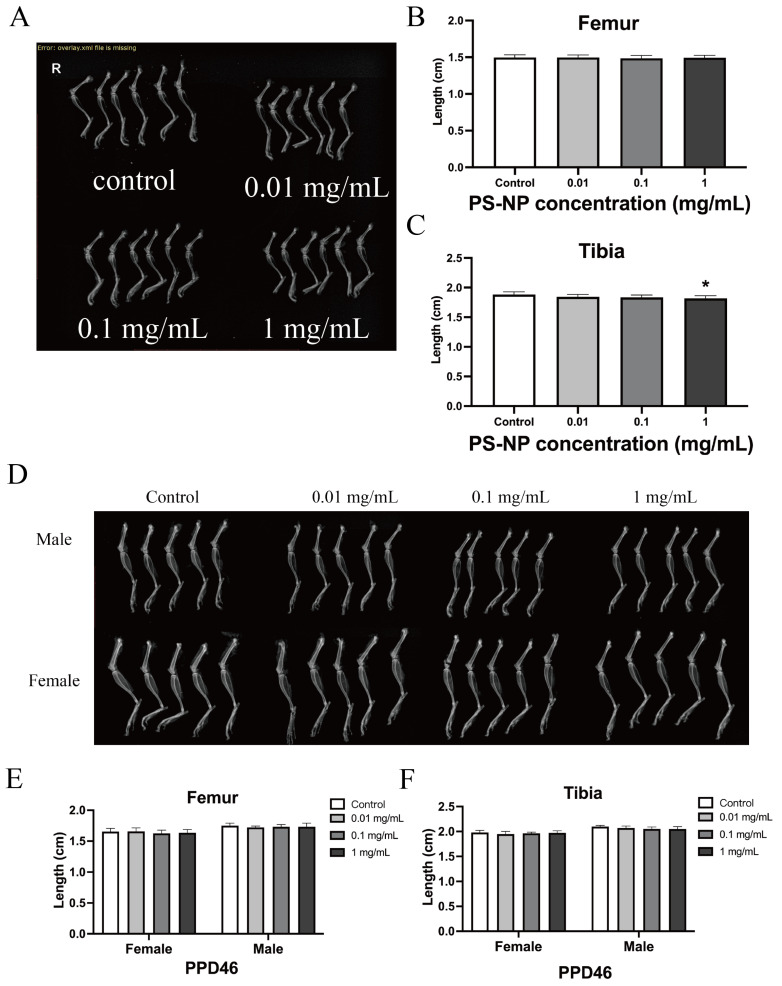
(**A**–**C**) Effects of different doses of PS-NP exposure on bone development of right hind limbs in offspring mice at PDD18 and PDD46. Asterisks indicate statistically significant differences. (**D**–**F**) Effects of different doses of PS-NP exposure on bone development of right hind limbs in offspring mice at PDD46. Data were analyzed using one-way ANOVA and are expressed as mean ± SD (n = 6).

At PDD18, we dissected the offspring mice to obtain their organs, including the heart, liver, spleen, lungs, kidneys, and brain. We assessed the organ weight and visceral body ratio and conducted histological evaluations. Exposure to PS-NPs at various doses resulted in delayed liver development in the lactating mice. Analysis of the heart, spleen, and kidneys revealed varying weight changes compared with their visceral body ratios, suggesting a need for further investigation. However, no significant differences in organ weight or visceral body ratio were observed for the lungs or brain across the dose groups (Figure 2 and Supplementary Figure S2), indicating that PS-NP exposure via drinking water did not significantly impact lung or brain development in the lactating mice. X-ray imaging of the right hind limb bones at PDD18 (Figure 3A–C) revealed no apparent differences in femur or tibia ossification. Nonetheless, the measurements showed a significant decrease in tibia length in the high-dose group compared with the control group (*p* < 0.05). This suggests that high-dose PS-NP exposure via drinking water may delay tibia development in lactating mice. Histological evaluations of the heart, liver, spleen, lungs, kidneys, and brain, detailed in Supplementary Figure S5, revealed no significant tissue damage, inflammatory lesions, or structural differences among the groups. This indicates that PS-NP exposure did not cause visible tissue damage or developmental abnormalities in the organs of the PDD18 offspring mice.

### 3.4. Impact of PS-NPs on Organ Development in PDD46 Offspring Mice

At PDD46, necropsies were performed on the offspring mice to obtain and evaluate organs including the heart, liver, spleen, lungs, kidneys, and brain. We assessed the organ weights and visceral body ratios and conducted histological analysis using H&E staining. As shown in Supplementary Figure S3, no significant differences in the organ weights or visceral body ratios were found for the heart, spleen, or lungs across the various PS-NP dose groups compared with the control group.

**Figure 4:**
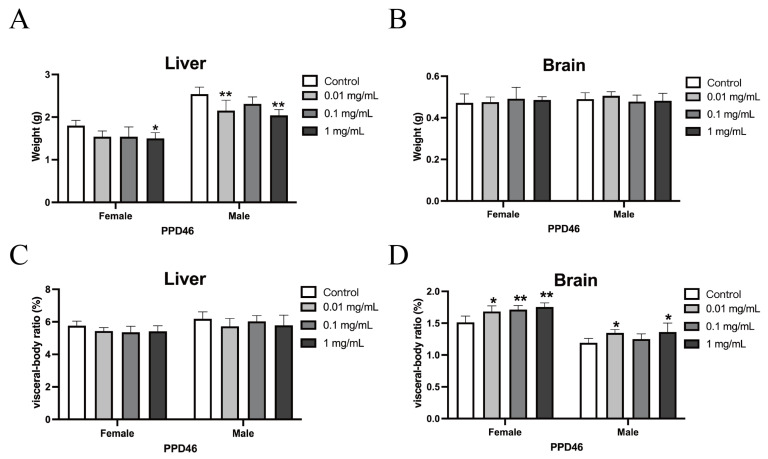
(**A**–**D**) Changes in liver and brain weights and visceral body ratio of PDD46 offspring mice after exposure to PS-NPs at different doses. Note: Compared with the control group, * *p* < 0.05; ** *p* < 0.01. Data were analyzed using one-way ANOVA and are expressed as mean ± SD (n = 5).

However, significant reductions in liver weight were observed in the high-dose female group compared with the control group (* *p* < 0.05) and in both the low- and high-dose male groups compared with the control, with highly significant differences (** *p* < 0.01). Despite these reductions, the visceral body ratio for the liver did not differ significantly. Additionally, significant reductions in kidney weight were noted in the high-dose male group compared with the control group (* *p* < 0.05), although the visceral body ratio for the kidneys did not show significant differences. There were no significant differences in the brain weights among the dose groups, but the low-dose female group, low-dose male group, and high-dose male group exhibited significant increases in the brain-to-body weight ratio compared with the control group (* *p* < 0.05), while the medium- and high-dose female groups showed highly significant increases (** *p* < 0.01), as shown in Figure 4.

The histological evaluations from H&E staining, detailed in Supplementary Figure S6, revealed no significant tissue damage or inflammatory changes in any of the organs. This indicates that PS-NP exposure did not lead to significant histopathological alterations in the liver, heart, spleen, lungs, kidneys, or brain of the PDD46 mice. X-ray imaging of the right hind limb skeletal structure (Figure 3D–F) showed no significant differences in the ossification of the femur or tibia. Measurements of the femur and tibia lengths from the X-ray images indicated no significant effects on bone length growth due to PS-NP exposure in either the female or male mice.

To assess the potential effects on liver and kidney function, we measured the serum levels of albumin (ALB) and cholesterol (CHOL) for liver function, alkaline phosphatase (ALP) and alanine aminotransferase (ALT) for liver damage, and blood creatinine (CREA) and blood urea (UREA) for kidney function. As shown in Supplementary Figure S4, no significant differences in these markers were observed among the groups, suggesting that PS-NP exposure did not induce significant liver or kidney toxicity or affect liver function in the early sexually matured mice.

### 3.5. Impact of PS-NPs on Intestinal Damage in Offspring Mice at Various Developmental Stages

**Figure 5:**
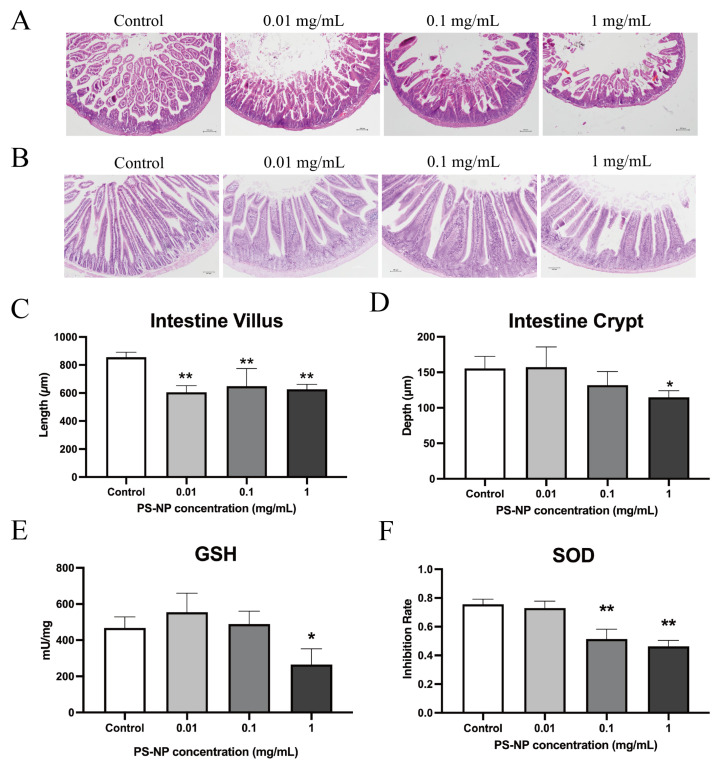
The intestinal damage observed in the offspring mice at different stages due to PS-NP exposure. (**A**,**B**) Histological examination of offspring mice intestinal tissues with H&E staining after exposure to different doses of PS-NPs. (**A**,**B**) PDD18 and PDD46, respectively. (**C**,**D**) The villus and crypt depths of the intestines in the PDD46 offspring mice. (**E**,**F**) Effects of different doses of PS-NPs on intestinal SOD and total GSH in offspring mice at PDD46 (n = 5). Note: Compared with the control group, * *p* < 0.05; ** *p* < 0.01.

Histological examination of the H&E-stained sections from the small intestines of the PDD18 and PDD46 offspring mice revealed dissolution and damage at the tips of the intestinal villi, along with significant shortening of the villi length in the low-, medium-, and high-dose PS-NP exposure groups compared with the control group (Figure 5A,B). The measurements, averaged from at least 10 villi or crypt depths per intestinal section across five samples per group, showed highly significant differences (** *p* < 0.01) in the villi length across all PS-NP exposure groups compared with the control group. The crypt depths were significantly reduced only in the high-dose group compared with the control group (* *p* < 0.05), with no significant differences observed in the other groups (Figure 5C,D).

Oxidative stress in the small intestine plays a critical role in disrupting epithelial cell renewal and homeostasis, directly influencing gut health [19]. To assess oxidative stress damage in the small intestines of the PDD46 offspring mice post exposure, we measured the superoxide dismutase (SOD) inhibition rates and total glutathione (Figure 5E,F). The SOD inhibition rates were significantly decreased (** *p* < 0.01) in the medium- and high-dose PS-NP exposure groups compared with the control group. The total GSH showed significant decreases (* *p* < 0.05) only in the high-dose exposure group, with no significant differences observed in the low- or medium-dose groups. Additionally, we used red fluorescent-labeled PS-NPs to prepare the frozen intestinal sections to observe PS-NP uptake (Supplementary Figure S7). PS-NPs were observed only in the mammary glands of the dams, liver, and intestines of the offspring.

**Figure 6:**
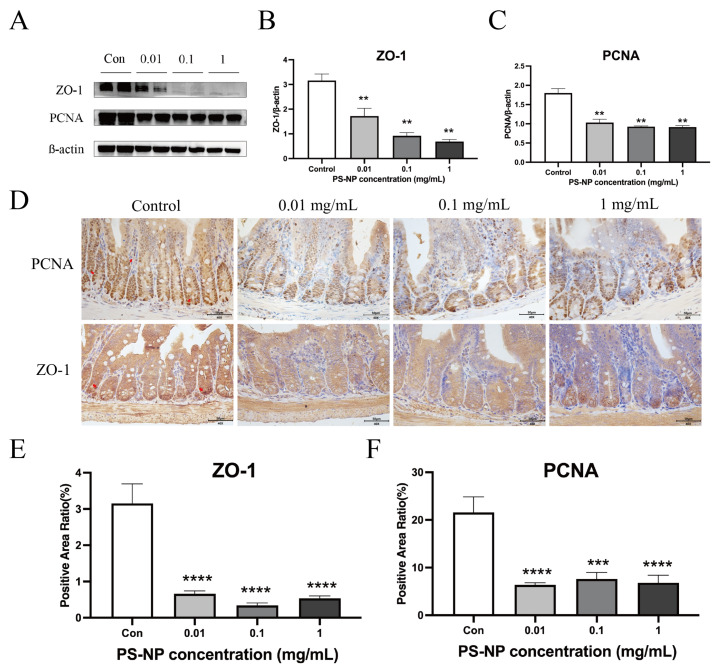
The intestinal damage observed in the offspring mice at different stages due to PS-NP exposure. (**A**–**C**) Effects of different doses of PS-NP exposure on intestinal tight junction protein ZO-1 and nuclear proliferation antigen PCNA in offspring mice of PDD46. (**D**–**F**) Immunohistochemistry of intestinal ZO-1 and PCNA in offspring mice at PDD46 after exposure to different doses of PS-NPs. Note: Compared with the control group, ** *p* < 0.01; *** *p* < 0.001; **** *p* < 0.0001.

To further evaluate the impact of PS-NP exposure on intestinal proliferation and barrier integrity, we performed western blot (WB) and immunohistochemistry analyses on the small intestines of the PDD46 offspring mice. We assessed the expression of proliferating cell nuclear antigen (PCNA) and the tight junction protein ZO-1. WB analysis indicated a highly significant decrease in ZO-1 expression in the low-, medium-, and high-dose PS-NP exposure groups compared with the control (** *p* < 0.01), with significant differences observed among the dose groups (* *p* < 0.05), suggesting dose-dependent ZO-1 downregulation. PCNA expression significantly differed (** *p* < 0.01) in all toxicity groups (low, medium, and high doses) compared with the control group, but no significant differences were observed among the toxicity groups (Figure 6A–C). Immunohistochemical evaluation confirmed these findings, showing reduced positive signals(red arrow in image) for PCNA (nuclear) and ZO-1 (cytoplasmic, membrane) in the PS-NP exposure groups compared with the control group (Figure 6D–F). In summary, exposure to sufficient doses of PS-NPs induced oxidative stress damage in the small intestines of the offspring mice.

## 4. Discussion

Nanoplastic pollution represents a significant global public health concern. Understanding its toxicity and mechanisms in mammalian models, particularly mice, is crucial for scientific progress [20]. This study aims to investigate the toxicological effects of nano-sized microplastics ingested through drinking water on the growth and development of juvenile mice. Polystyrene nanoparticles (PS-NPs) with a size of 100 nm were used at various concentrations to minimize the stress associated with the gavage procedures. Regarding the dose selection, we referenced current research on microplastic contamination in food and drinking water, which reports that the maximum human exposure via oral intake is approximately 398–431 g per year per person [11], while the minimum value is roughly 33.2 g per year per person [12]. The maximum and minimum exposure levels reported in the existing literature were normalized using the body surface area method and then applied to establish the high-dose (1 mg/mL) and medium-dose groups (0.1 mg/mL) for the experiments. A recent review noted that some studies used maximum concentrations exceeding 1000 mg/kg [21]. These variations highlight that current assessments often miss the multiple exposure routes and limitations in detection methods, emphasizing the need for high-concentration exposures to reflect real-world scenarios. Furthermore, exploring the thresholds for adverse effects is critical [22,23]. Additionally, a toxicity study on pregnant mice recommended a maximum dose of 1 mg/day via drinking water, corresponding to 100 µg/mL, which aligns with our medium dosage [17]. To address concerns that microplastic toxicity studies may deviate from real-world environmental relevance, we also established a low-dose group with even lower exposure levels (0.01 mg/kg). A chronic study used a lower dose of 10 µg/mL, corresponding to our lowest dose, but focused on chronic toxicity over 32 weeks [24]. Based on the above discussion, we believe that the dose groups established in this study may encompass the range of microplastic exposure levels humans might encounter through oral ingestion, thereby providing a better simulation of the potential harms of microplastic exposure on pregnant animals and the early development of their offspring.

We assessed the mice at PDD18 and PDD46, with PDD18 marking the end of lactation and when the mice began drinking water and eating independently [25]. This timing allowed us to observe the effects of PS-NPs through drinking water more clearly. Exposure to PS-NPs resulted in weight suppression across all dose groups. During lactation, weight suppression ranged from 4.8% to 14.1% of the normal body weight. At early sexual maturity, the female mice showed weight suppression ranging from 9.3% to 11.1%, while the male mice experienced a reduction of 8.5–13.7%. These findings align with previous studies which reported inhibited weight growth due to microplastic exposure [26]. The dose range in our study spanned from 0.01 mg/mL to 1 mg/mL, with the lowest dose being 0.01 mg/mL, which is lower than the 1 mg/day toxic dose reported in earlier studies [17]. This lower dose is more reflective of natural microplastic pollution scenarios, and we chose this range to ensure that our experimental doses were more environmentally relevant. The weight suppression observed in the medium-dose group at early sexual maturity, though noticeable, did not reach statistical significance, which may be attributed to factors such as inherent statistical variability, or the potential nonlinear relationship between dose and toxicity. The lack of statistical significance may also suggest that the weight changes could be due to factors other than microplastic exposure, such as variations in water intake or other environmental conditions. Garcia et al. found that orally ingested polystyrene or mixed polymer microspheres can cross the intestinal barrier, accumulate in tissues such as the brain, liver, and kidneys, and induce concentration-dependent and polymer type-specific metabolic changes [27].In our study, liver development was affected by PS-NPs, evidenced by a reduced liver weight and proportion relative to the body weight, though the liver function indicators and histological examinations did not show significant impairments. This suggests that PS-NPs may delay liver development rather than directly cause liver toxicity, consistent with studies indicating microplastics can induce oxidative damage in fish livers without significant histological damage [28]. Delayed liver development could stem from broader impacts on growth metabolism. The kidneys also showed reduced weights in the high-dose and male mice, though no significant functional impairment was observed. The brain weight relative to the body weight showed an upward trend, but histological examination did not reveal significant abnormalities. Some studies suggested that nanoplastics can affect the brain, but our findings showed no significant differences in the absolute brain weight, with increased ratios likely related to inhibited weight gain [29]. The neurotoxic effects in other studies included reduced expression of neurotransmitter-related genes [30]. Overall, while PS-NPs did not cause significant tissue damage or functional abnormalities in most vital organs, the results highlight the need for more sensitive monitoring methods and further exploration at the cellular level to fully understand their effects.

The most significant damage caused by PS-NPs in the offspring mice was observed in the small intestines, consistent with previous environmental toxicity studies [31]. However, no inflammation or related intestinal pathologies were detected in this study. The observed toxic effects of PS-NPs on the small intestine suggest impaired intestinal health and function, including (1) dissolution and damage of the villi at the apex; (2) inhibition of crypt cell proliferation, as indicated by decreased PCNA expression; (3) disruption of intestinal tight junctions, evidenced by the reduced expression of tight junction proteins such as ZO-1; and (4) induction of oxidative damage. These findings may indicate compromised nutrient absorption and intestinal barrier function. Oxidative stress in the intestinal epithelium is closely linked to alterations in gut barrier integrity and epithelial cell renewal, which are both critical factors in gut health [32]. There was a study which demonstrated that PS-MPs cause intestinal barrier dysfunction through the ROS-dependent NF-κB/NLRP3/IL-1β/MLCK pathway [33]. Thus, in our study, we specifically focused on the small intestine for oxidative stress analysis, considering its role as the primary site of exposure to orally ingested nanoplastics. Furthermore, localized oxidative damage in the small intestine can initiate systemic effects, making it a suitable model for studying the early impacts of nanoplastic exposure [34]. These findings suggest that the shortened villi in the PS-NP-exposed mice resulted from oxidative damage, epithelial cell necrosis, disrupted tight junctions, and inhibited crypt cell proliferation. Intestinal villi play a crucial role in nutrient absorption, and their condition, along with the crypts and mucosal integrity, significantly impacts growth, development, and overall health in livestock and poultry [35,36]. The combined effects of these factors lead to intestinal damage and impaired nutrient absorption in mice, resulting in developmental suppression and abnormal organ weights, as observed in this study. Additionally, the impact of microplastics on gut microbiota was not explored in this study, unlike in other research [37]. Our study focused on developmental mice, where intestinal damage primarily affected the small intestine, leading to impaired nutrient absorption and delayed growth and development in offspring.

Although no obvious functional toxicity was observed in the early development of juvenile mice due to microplastic exposure in this study, microplastic pollution is not merely a matter of current contamination but also an issue of incremental accumulation. The microplastic release from infant feeding bottles in different regions varies widely, ranging from 14,600 to 4,550,000 particles per person per day [38]. The level of microplastic exposure in infants is much higher than was previously recognized. Additionally, the developmental process in infants is significantly longer than that in mice, resulting in a longer exposure time to microplastics. Future research should focus more closely on the long-term effects of microplastic exposure on the development of infants and young children.

## 5. Conclusions

Exposure to PS-NPs during both the lactation (PDD18) and juvenile (PDD46) periods resulted in delayed weight gain in mice, which may have implications for the development of vital organs such as the liver and kidneys. Although no functional abnormalities or overt signs of toxicity were observed, these exposures led to significant alterations in the growth and development of the small intestine. Notably, intestinal villi were shortened, tight junction integrity was disrupted, epithelial proliferation was suppressed, and oxidative stress responses were activated. These findings suggest that PS-NPs could have a detrimental impact on intestinal health, potentially impairing nutrient absorption and overall gut function. Furthermore, these results contribute to our understanding of nanoplastic toxicity, particularly in the context of early life exposure, where such changes may have long-term consequences for organ development and function. By highlighting these effects, this study underscores the importance of evaluating environmental nanoplastic exposure, particularly in vulnerable populations such as infants and children, whose developing systems may be more susceptible to these toxic effects.

## Data Availability

The raw data supporting the conclusions of this article will be made available by the authors on request.

## Supplementary Materials

The following supporting information can be downloaded at: https://www.mdpi.com/article/10.3390/nano15010069/s1.

## Abbreviations

The following abbreviations are used in this manuscript:

PS-NPs	Polystyrene nanoplastics
MPs	Microplastics
NPs	Nanoplastics
GABA	Gamma-aminobutyric acid
DLS	Dynamic light scattering
FTIR	Fourier transform infrared spectroscopy
OCT	Optimal cutting temperature compound
DAPI	4′,6-diamidino-2′-phenylindole
DAB	Diaminobenzidine
RIPA	Radio-immunoprecipitation assay
PMSF	Protein protector phenylmethanesulfonyl fluoride
BCA	Bicinchoninic acid assay
GSH	Glutathione
PDD	Post delivery day
PCNA	Proliferating cell nuclear antigen
ALB	Albumin
CHOL	Cholesterol
ALP	Alkaline phosphatase
ALT	Alanine aminotransferase
SOD	Superoxide dismutase
WB	Western blot
