# Supplementary material for: Intestinal Barrier Damage and Growth Retardation Caused by Exposure to Polystyrene Nanoplastics Through Lactation Milk in Developing Mice
Source: Nanomaterials (Basel). 2025 Jan 4;15(1):69. doi: 10.3390/nano15010069 (PMC11722969; doi:10.3390/nano15010069)
Supplement: Supplementary file 1 [file nanomaterials-15-00069-s001.zip › nanomaterials-3369265-supplementary.pdf]

Intestinal Barrier Damage and Growth Retardation Caused by Exposure to Polystyrene Nanoplastics Through Lactation Milk in Developing Mice

Chaoyu Zhou [1,2,†], Haiyan Wu [1,†], Lei Zhang [2], Xiao Xiao [2], Xiaodan Wang [2], Mingju Li [3], Runqiu Cai [1], Jia You [4], Qi Chen [5], Yifei Yang [1], Xinyuan Tian [1], Qianyu Bai [1], Yinzhu Chen [1], Huihui Bao [2,*] and Tianlong Liu [1,*]

1 National Key Laboratory of Veterinary Public Health and Safety, College of Veterinary Medicine, China Agricultural University, Beijing 100193, China
2 Chinese Academy of Medical Science Research Unit, NHC Key Laboratory of Food Safety Risk Assessment,China National Center for Food Safety Risk Assessment, Beijing 100022, China
3 Yantai Animal Disease Control Center, Yantai 264003, China
4 Yantai Agricultural Technology Extension Center, Yantai 264001, China
5 Livestock and Veterinary Development Center of Zoucheng, Hong Kong, China
* Correspondence: baohuihui@cfsa.net.cn (H.B.); liutianlong@cau.edu.cn (L.L.)
† These authors contributed equally to this work.

Corresponding Author:
Huihui Bao
Chinese Academy of Medical Science Research Unit, NHC Key Laboratory of Food Safety Risk Assessment,China National Center for Food Safety Risk Assessment, Beijing 100022, China
baohuihui@cfsa.net.cn;
&
Tianlong Liu
National Key Laboratory of Veterinary Public Health and Safety, College of Veterinary Medicine, China Agricultural University, Yuanmingyuan West Road, Haidian District, Beijing 100193, China
liutianlong@cau.edu.cn

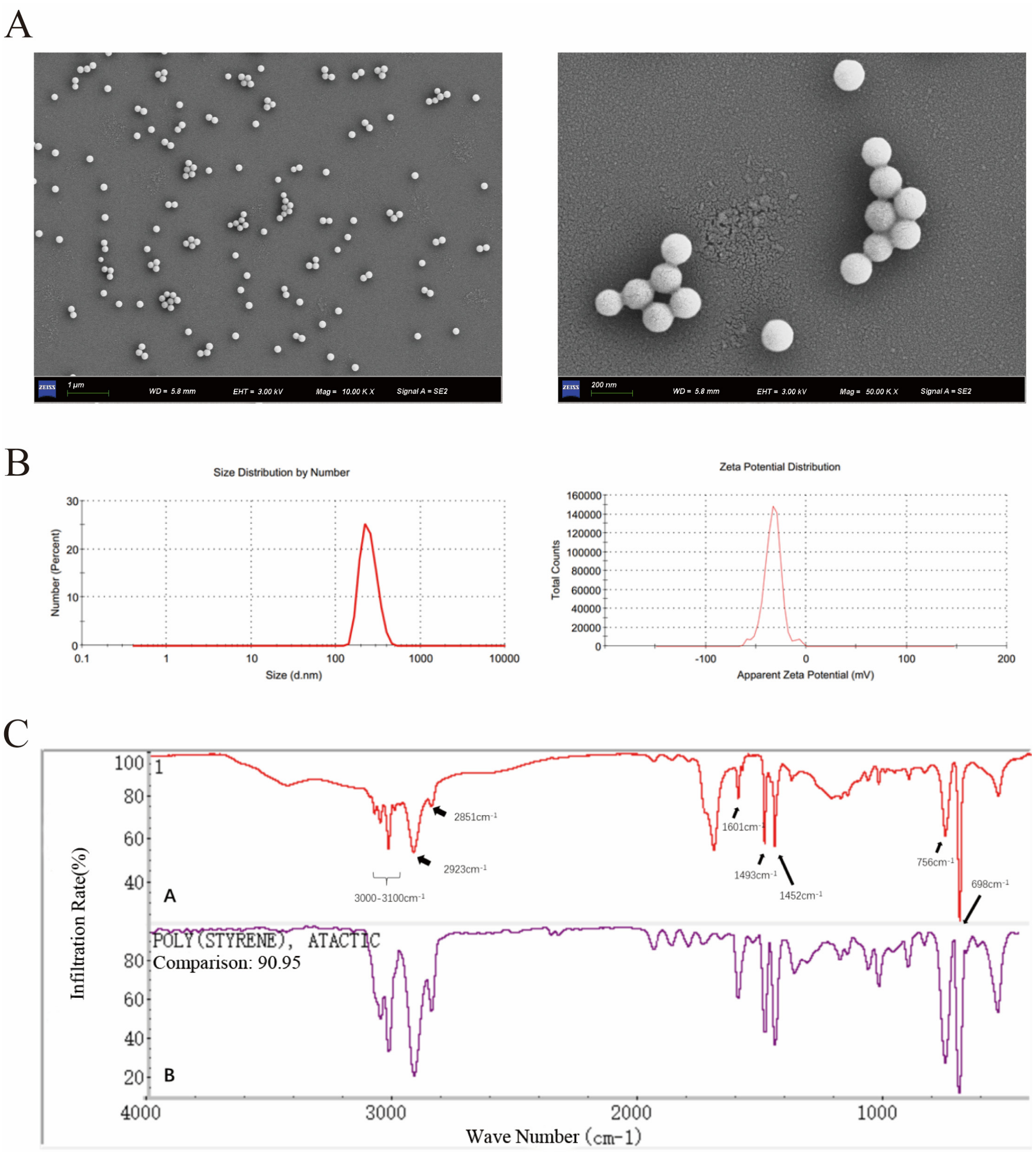

Supplementary Figure S1. Characterization of PS-NPs used in this study. A, PS-NPs' images of low-magnification and high-magnification of view under SEM, respectively. B, Hydrated particle size and Zeta potential of PS-NPs. C, Fourier transform infrared spectrum of PS-NPs.

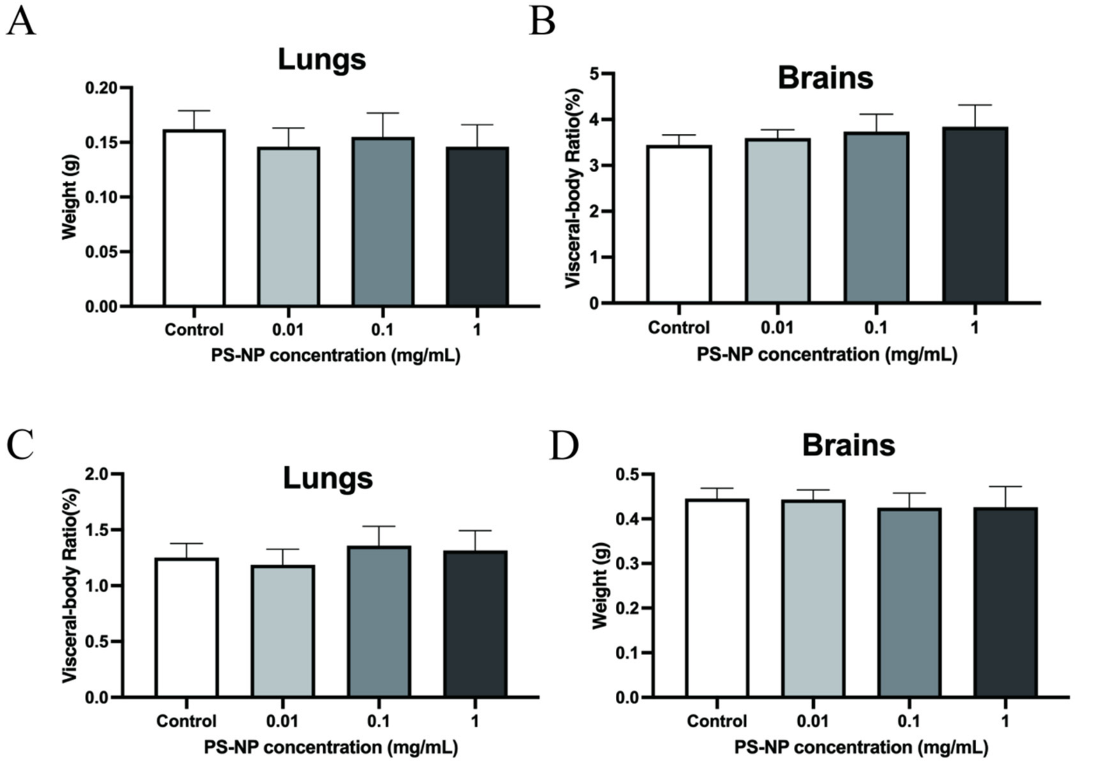

Supplementary Figure S2. The effects of PS-NPs exposure on the organs of PDD18 (lactation period) offspring mice. (A, B) Changes of the main organ weights in offspring mice of PDD18 after exposure to different doses of PS-NPs(n=10). (C, D) Changes of the main organ visceral-body ratio in offspring mice of PDD18 after exposure to different doses of PS-NPs. Data were analyzed using one-way ANOVA and expressed as mean ± SD (n=10).

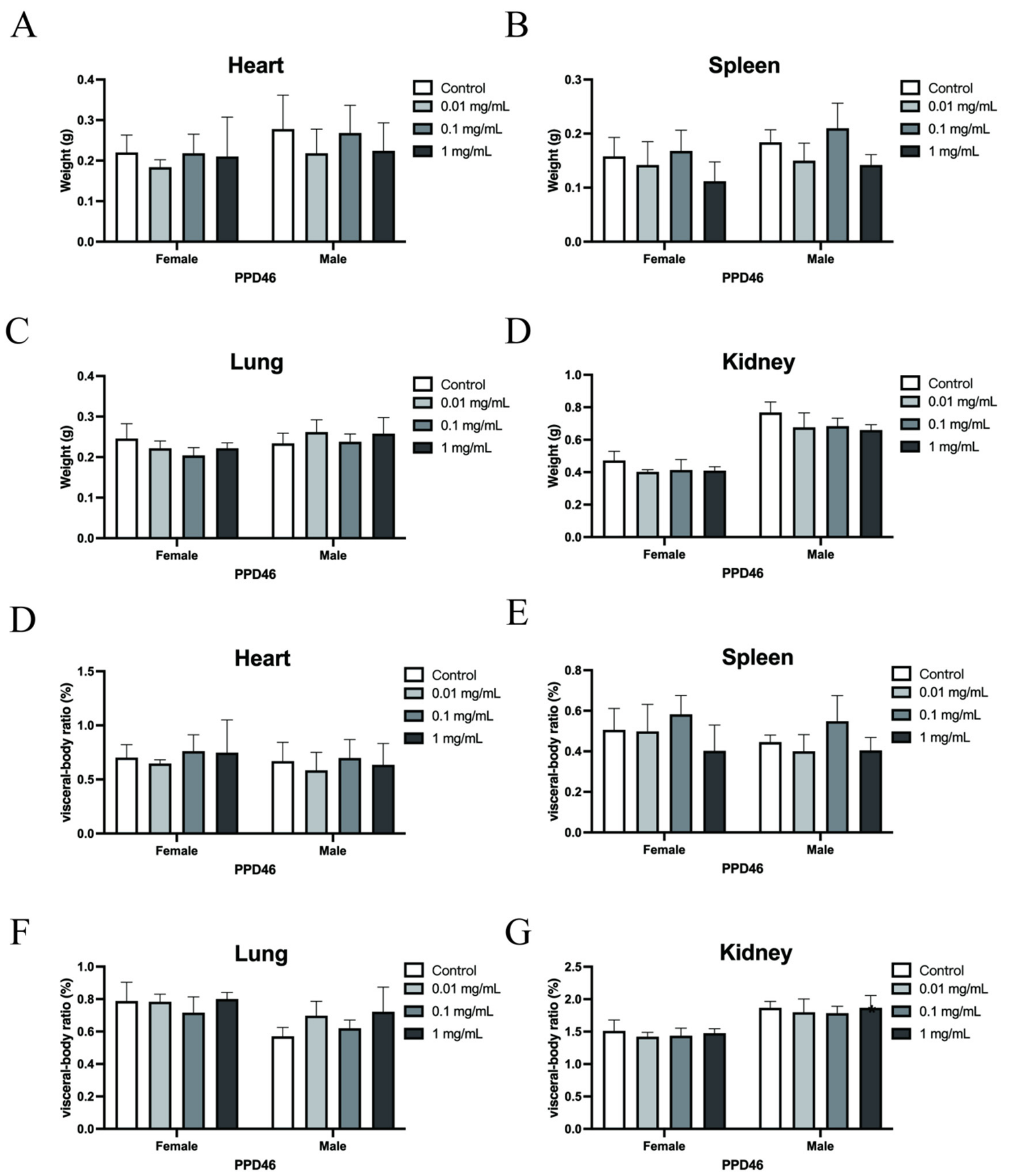

Supplementary Figure S3. The effects of PS-NPs exposure on the organs of PDD46 offspring mice. (**A-D**) Changes of the main organ weights in offspring mice of PDD46 after exposure to different doses of PS-NPs(n=5). (D-G) Changes of the main organ visceral-body ratio in offspring mice of PDD46 after exposure to different doses of PS-NPs. Data were analyzed using one-way ANOVA and expressed as mean ± SD (n=5).

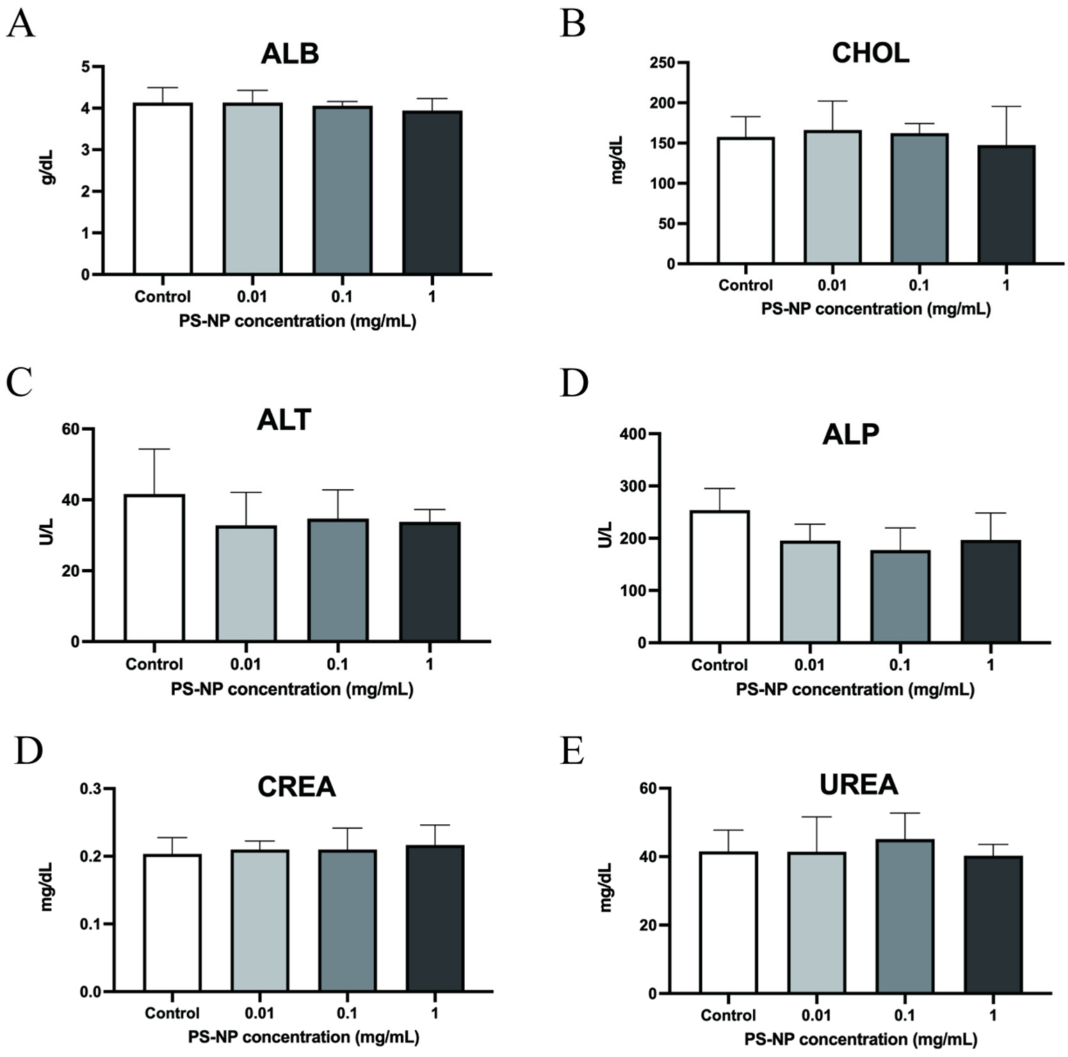

Supplementary Figure S4. (A-E) Effects of different doses of PS-NPs exposure on serum ALB, CHOL, ALP, ALT, CREA and UREA of PDD46 offspring mice. Data were analyzed using one-way ANOVA and expressed as mean ± SD (n=5).

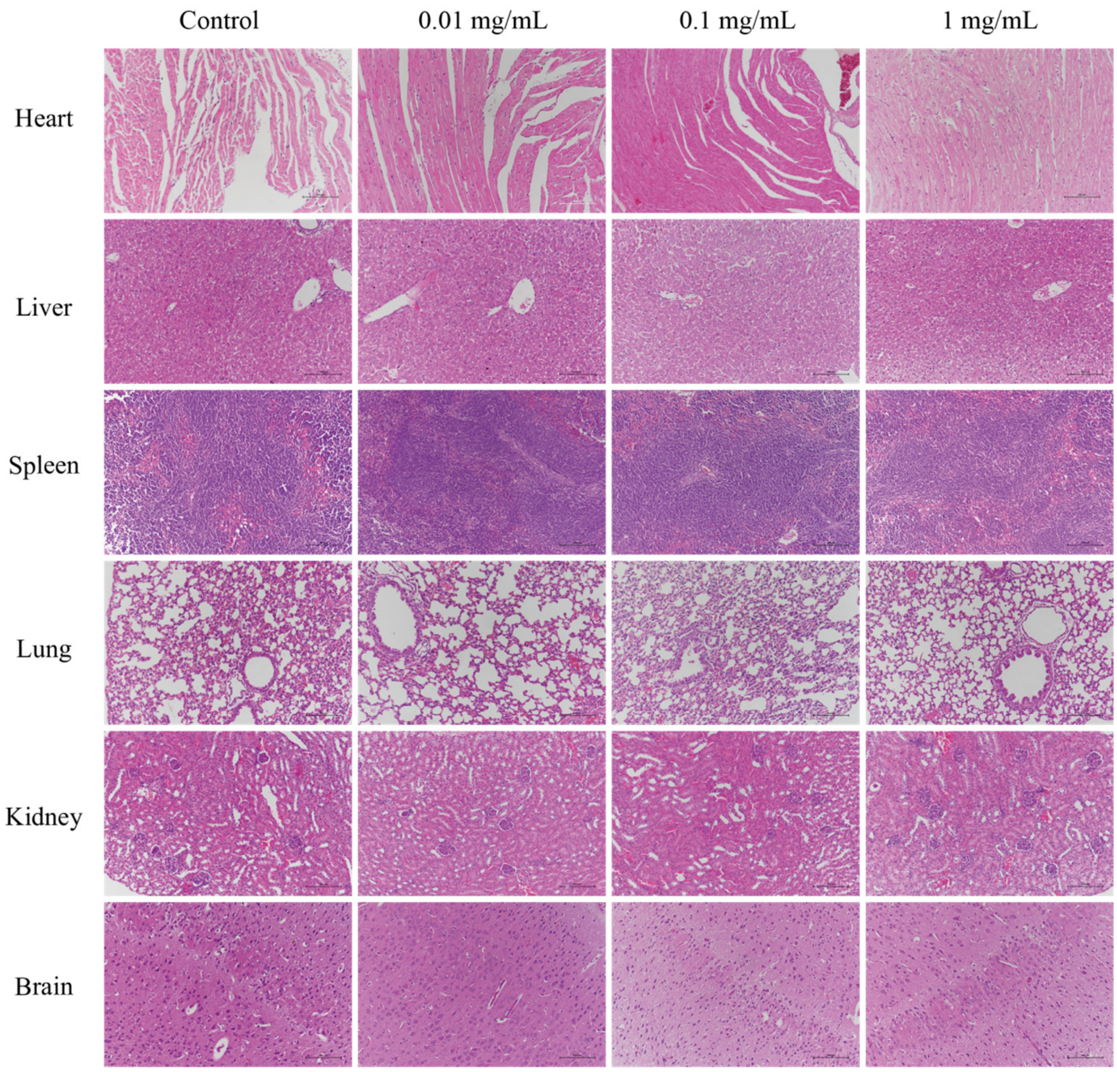

Supplementary Figure S5. Hematoxylin and eosin staining of vital organs in mice treated with nano-PS in offspring mice of PDD18(n=10).

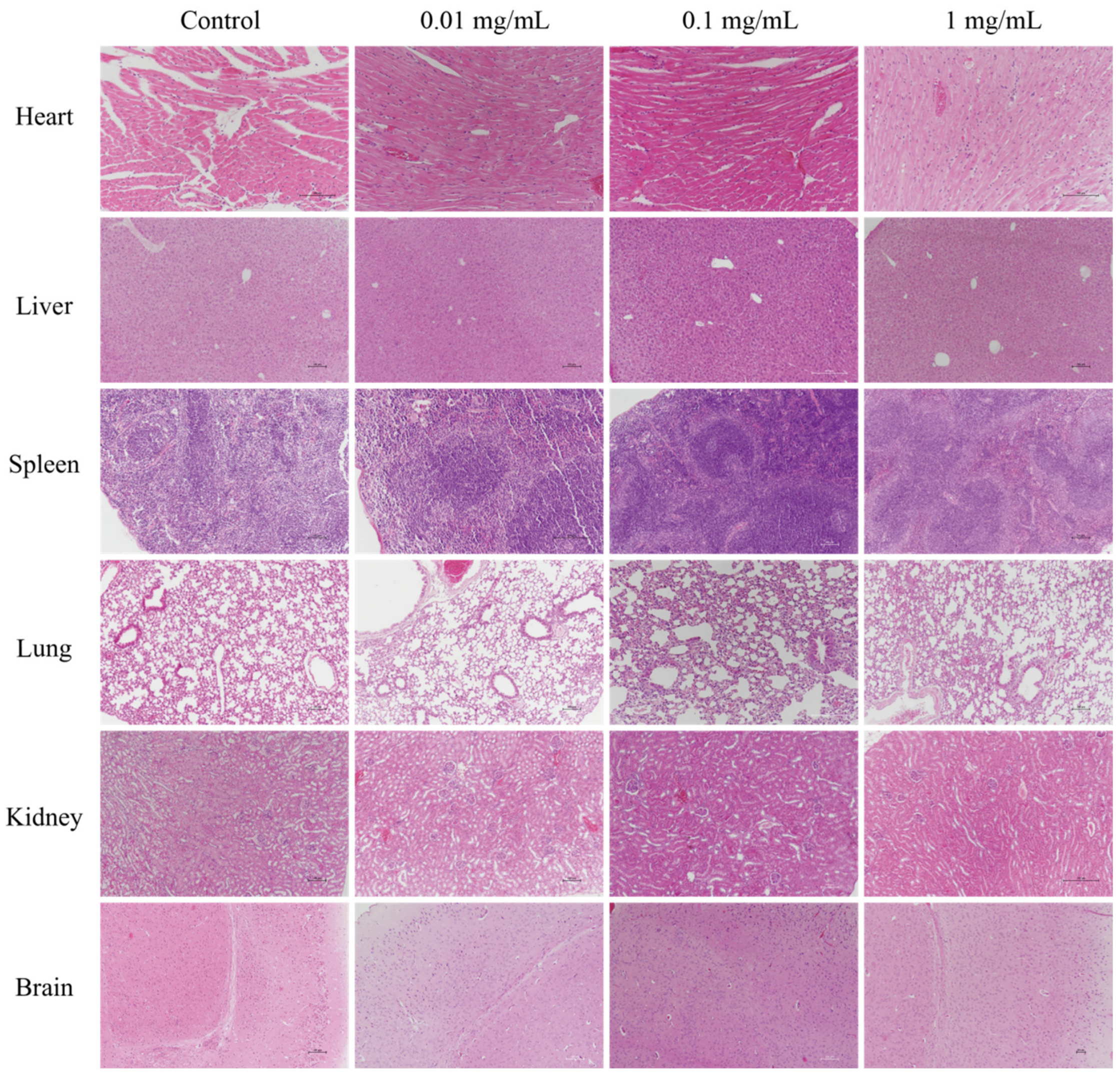

Supplementary Figure S6. Hematoxylin and eosin staining of vital organs in mice treated with nano-PS in offspring mice of PDD46(n=5).

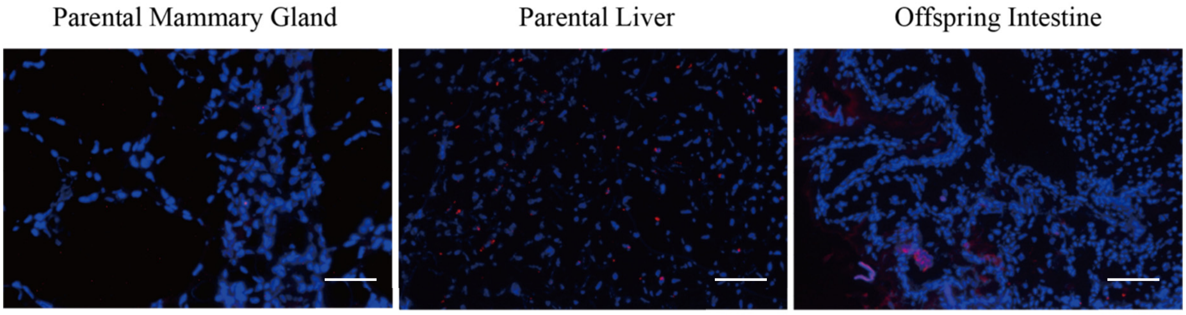

Supplementary Figure S7. PS-NPs in mouse tissues, with blue indicating DAPI staining and red representing Rhodamine B-labeled PS-NPs. Observations reveal the presence of PS-NPs in the mammary glands, liver of lactating dams, and the intestines of offspring that were not directly exposed to PS-NPs through drinking water. This

suggests that PS-NPs ingested by the dams through drinking water can potentially be transferred to the offspring via breast milk(bar =20 μm).
